# Argonaute proteins from human gastrointestinal bacteria catalyze DNA-guided cleavage of single- and double-stranded DNA at 37 °C

**DOI:** 10.1038/s41421-019-0105-y

**Published:** 2019-07-30

**Authors:** Yuanwei Cao, Wen Sun, Jinfeng Wang, Gang Sheng, Guanghai Xiang, Tongtong Zhang, Wenyu Shi, Chun Li, Yanli Wang, Fangqing Zhao, Haoyi Wang

**Affiliations:** 1 State Key Laboratory of Stem Cell and Reproductive Biology, Institute of Zoology, Chinese Academy of Sciences, 100101 Beijing, China; 2 University of Chinese Academy of Sciences, 100049 Beijing, China; 3 Institute for Stem Cell and Regeneration, Chinese Academy of Sciences, 100101 Beijing, China; 4 Beijing Institutes of Life Science, Chinese Academy of Sciences, 100101 Beijing, China; 5 Key Laboratory of RNA Biology, CAS Center for Excellence in Biomacromolecules, Institute of Biophysics, Chinese Academy of Sciences, 100101 Beijing, China; 6 Institute of Microbiology, Chinese Academy of Sciences, 100101 Beijing, China; 7 Hebei University, Baoding, 071002 Hebei, China; 8 Collaborative Innovation Center of Genetics and Development, 200438 Shanghai, China; 9 Center for Excellence in Animal Evolution and Genetics, Chinese Academy of Sciences, 650223 Kunming, Yunnan China

**Keywords:** Biological techniques, Bioinformatics

Dear Editor,

Eukaryotic Argonaute (eAgos) proteins are the key players in RNA interference (RNAi) pathways by functioning as an RNA-guided RNA endonucleases^1^. The homologous prokaryotic Argonaute proteins (pAgos) are functionally versatile, some of which can target DNA guided by cognate small DNA. However, most of the well-characterized pAgos, such as the ones from *Thermus thermophilus* (TtAgo)^2^, *Pyrococcus furiosus* (PfAgo)^3^, and *Methanocaldococcus jannaschii* (MjAgo)^4^, are derived from thermophiles and function optimally at temperatures above 65 °C, making them unlikely to be utilized as a genome-editing tool in mesophilic organisms.

In searching for pAgos that could function at human physiological temperature, we performed searches using PFAM hidden Markov model profiles to identify PIWI (P-element-induced wimpy testis)-containing proteins from published human microbiome data^5,6^, and identified dozens of putative Ago genes from several typical human gut microbes, including *Bacteriodes*, *Clostridium* and *Intestinibacter* (Supplementary Table S1). Ago proteins from *Clostridium perfringens* (CpAgo) and *Intestinibacter bartlettii* (IbAgo, Synonym, *Clostridium bartlettii*) contain an N (N-terminal) domain, a PAZ (PIWI-Argonaute-Zwille) domain and a MID (middle) domain, along with two domain linkers, L1 and L2, representing a typical full-length Ago structure. Phylogenetic analysis revealed that CpAgo and IbAgo are closely related (Fig. 1a). Detailed sequence alignments showed that both CpAgo and IbAgo have conserved catalytic tetrad (DEDD or DEDH) in the PIWI domain, indicating that they may be catalytically active (Supplementary Fig. S1a).

**Fig. 1:**
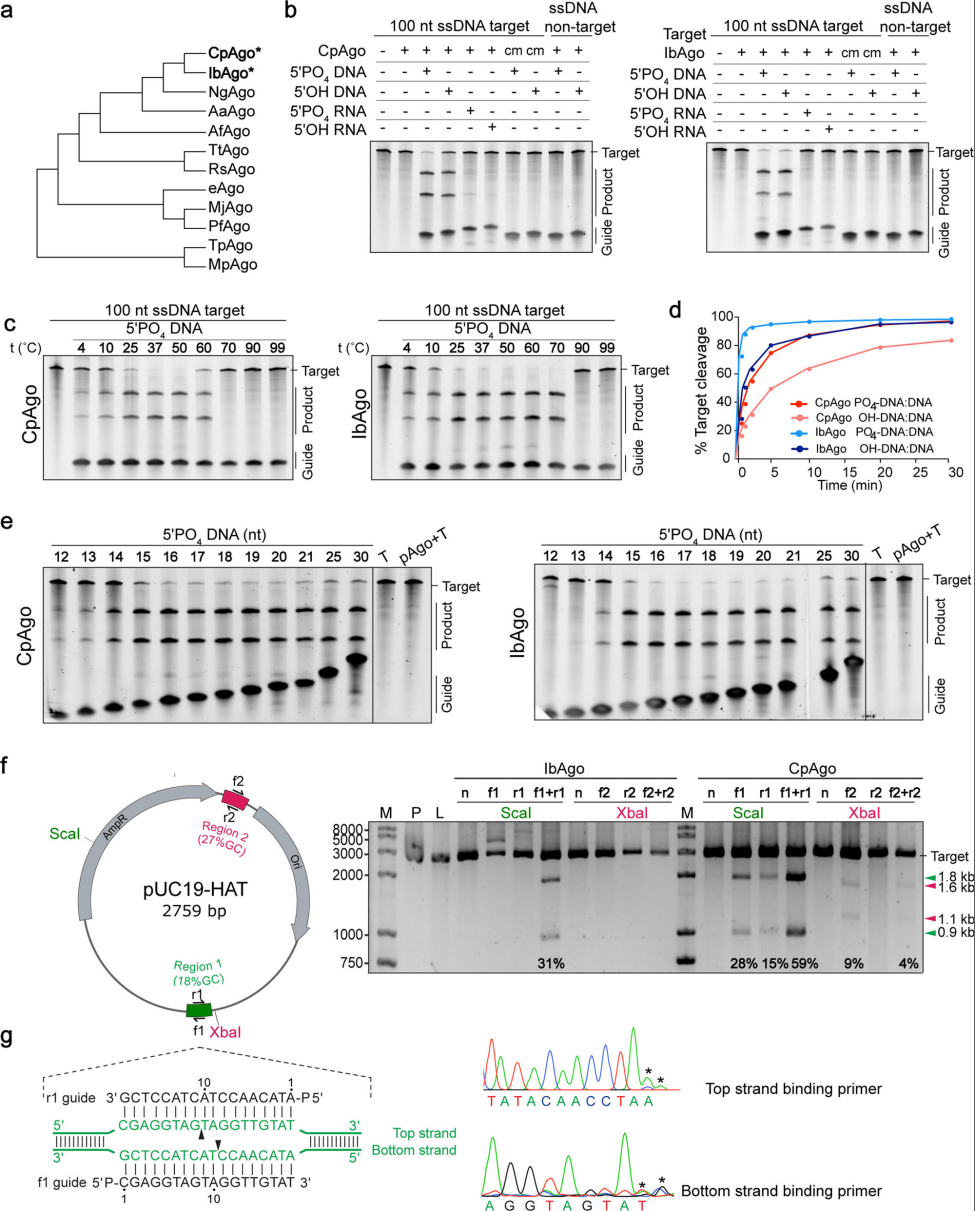
Characterization of the DNA cleavage activity of CpAgo and IbAgo. **a** Schematic phylogenetic tree of CpAgo, IbAgo, and other previously characterized pAgos. **b** CpAgo and IbAgo cleave 100 nt single-stranded DNA (ssDNA) or non-target ssDNA with the 5’-P DNA or 5’-OH complementary DNA guides at 37 °C. cm: catalytically mutant. CpAgo or IbAgo were premixed with various guides for 30 min, followed by ssDNA cleavage in a 5:5:1 molar ratio (pAgo:guide:target) for 1 h. Products were resolved on a 15% denaturing polyacrylamide gel. Results are representative of three independent experiments. **c** Effect of temperature on CpAgo and IbAgo cleavage of 100 nt ssDNA. **d** Cleavage kinetics of 100 nt ssDNA substrate using 5’-P or 5’-OH DNA-guided CpAgo and IbAgo. **e** Effect of DNA guide lengths (12–21, 25, 30 nts) on ssDNA cleavage efficiency. T: 100 nt ssDNA target. **f** Double-stranded plasmid DNA cleavage by IbAgo and CpAgo at 37 °C. Pre-incubated IbAgo- and CpAgo-5’-P gDNA complex targeting two separate sites of plasmid pUC19-HAT were incubated with the plasmid at 37 °C for 2 h. The purified cleaved products were then digested with *Sca*I (for region 1) or *Xba*I (for region 2) for 2 h, followed by 2% agarose gel electrophoresis. Results are representative of three independent experiments. The reaction buffer for CpAgo contains 20 mM Tris-HCl pH 7.5, 50 mM NaCl, 2 mM DTT, 0.15 mM MnCl_2_. The reaction buffer for IbAgo contains 20 mM Tris-HCl pH 7.5, 50 mM NaCl, 2 mM DTT, 2.5 mM MnCl_2_. The percent cleavage is shown below each lane. M: DNA ladder. P: supercoiled plasmids. L: linearized plasmid. n: a pair of guides without sequence complementarity with target sequence. f1/f2: forward guide. r1/r2: reverse guide. (f1 + r1)/(f2 + r2): forward and reverse guide. **g** Sanger sequencing analysis of pUC19-HAT cleavage products by CpAgo using paired gDNAs (f1 + r1). The position of cleavage site is indicated by the termination of primer extension in the sequencing reaction. Sequencing artifacts are shown with an asterisk above the corresponding peaks

We purified CpAgo and IbAgo as well as their catalytic mutants (CM) (Supplementary Fig. S1b), and performed in vitro cleavage assay. We used 18 nucleotide (nt) RNA or DNA guides containing a 5’-P or 5’-OH group for guide-dependent RNA or DNA target cleavage (Supplementary Table S2). CpAgo and IbAgo could cleave 100 nt single-stranded DNA (ssDNA) utilizing both 5’-P and 5’-OH complementary DNA guides at 37 °C (Fig. 1b). Interestingly, ssDNA cleavage by either CpAgo or IbAgo with 5’-OH DNA guides resulted in a slight shift of the cleavage site compared with cleavage using 5’-P DNA guide. The sequencing results showed that ssDNA cleavage using 5’-P DNA guides occurs at the position between nucleotides 10 and 11 on the guide strand (Supplementary Fig. S2a), which is the canonical cleavage pattern for pAgos^2,3^. For 5’-OH DNA guide, however, cleavage occurs between nucleotides 11 and 12 on the guide strand (Supplementary Fig. S2b). A cleavage pattern shift was also observed when 5’-OH RNA guide was used together with MpAgo and human Ago2 (hAgo2) to cleave substrate DNA (for MpAgo) or RNA (for hAgo2)^7,8^. Substitutions of the third catalytic tetrad residues DEDX (D614A in CpAgo and D586A in IbAgo) completely abolished the guide-dependent ssDNA cleavage activity, indicating the cleavage is PIWI domain dependent (Fig. 1b). Both CpAgo and IbAgo function over a wide range of temperatures (4–70 °C for IbAgo, 4–60 °C for CpAgo) and they performed optimally at 37 °C (Fig. 1c). We also detected DNA guide-dependent RNA-targeting cleavage by CpAgo, with 5’-P guide being more efficient than 5’-OH guide (Supplementary Fig. S3).

We then tested substrate cleavage in the presence of different divalent metal ions, an essential requirement for Ago activity^9^. Substrate cleavage with different divalent metal ions (Mg^2+^, Ca^2+^, Mn^2+^, Fe^2+^, Co^2+^, Ni^2+^, Cu^2+^, and Zn^2+^) showed that both CpAgo and IbAgo could utilize Mg^2+^ and Mn^2+^ as cation, with Mn^2+^ supporting higher activity (Supplementary Fig. S4a). Both CpAgo and IbAgo are active in buffers with NaCl concentration varying from 50 to 250 mM, whereas higher NaCl concentration reduces activity (Supplementary Fig. S4b). pH does not have significant influence on DNA cleavage by CpAgo within the tested range (7.0–8.0) (Supplementary Fig. S4c). Using the optimized condition (20 mM Tris-HCl pH 7.5, 50 mM NaCl, 2 mM DTT, 5 mM MnCl_2_), cleavage kinetics were obtained with either 5’-P or 5’-OH guide DNA (gDNA) targeting cognate ssDNA targets. IbAgo has faster reaction rate than CpAgo, with a 72% cleavage efficiency using 5’-P guide after only 0.5 min, while comparable cleavage was obtained after 5 min for CpAgo (Fig. 1d). The Electrophoretic mobility shift assay (EMSA) showed that both CpAgo and IbAgo bind to 5’-P DNA guide more efficiently than 5’-OH guide, consistent with the cleavage data (Supplementary Fig. S5).

We next tested CpAgo and IbAgo cleavage efficiency using gDNA of different lengths, revealing that gDNA between 15 and 30 nt long resulted in high cleavage efficiency (Fig. 1e). In contrast to eAgos and some other pAgos^10,11^, we did not find any 5’ end nucleotide preference for either CpAgo or IbAgo, as all guides tested led to similar cleavage efficiency (Supplementary Fig. S6). The flexibility of length and 5’ end nucleotide of gDNA suggest minimal sequence restriction. To characterize the specificity, we tested the effect of gDNA-target mismatches on cleavage efficiency. Single mismatch at nucleotides 12–15 and 14–16 substantially reduced the DNA cleavage activity of CpAgo and IbAgo, respectively, whereas single mismatches at other positions were well-tolerated (Supplementary Fig. S7). These results suggest that the complementarity between gDNA and target at the guide 3’ region is essential for the DNA cleavage of CpAgo and IbAgo.

Given that both CpAgo and IbAgo have the catalytic tetrad DEDD and can cleave ssDNA target efficiently at 37 °C, we tested whether double-stranded DNA could be cleaved with a pair of guides each targeting one strand. We cloned the 100 nt AT-rich sequence (18% GC content) into pUC19 vector to serve as the target plasmid (pUC19-HAT). Impressively, target cleavage efficiency is up to 59% (CpAgo) and 31% (IbAgo) at 37 °C using pre-assembled pAgo with perfectly complementary 5’-P gDNA pair (f1 and r1) followed by *Sca*I digestion (Fig. 1f). The optimal Mn^2+^ concentrations used for CpAgo (0.15 mM) and IbAgo (2.5 mM) was determined by testing plasmid cleavage efficiency under various concentrations (Supplementary Fig. S8). Interestingly, CbAgo also cleaved the plasmid when only one guide was used, with the forward guide supporting better efficiency (28% and 15% for f1 and r1, respectively). Another pair of gDNAs (f2 and r2) also led to targeted cleavage of the plasmid at a different position (27% GC content, 100 bp window size centered on the guide sequence position), albeit with a lower efficiency (Fig. 1f). Sequencing results showed that the cleavage of both the top and bottom strand occurred at the position between nucleotides 10 and 11 on the guide strand, consistent with the ssDNA substrate cleavage using 5’-P gDNA (Fig. 1g). We also sequenced the cleavage products by CbAgo using single guide (f1) and found the bottom strand cleavage is canonical (nucleotide 10/11 of the guide strand), while the top strand cleavage occurred at nucleotide 15/16 of the guide strand (Supplementary Fig. S9). As no corresponding gDNA was used for top strand cleavage, the detailed mechanism of plasmid cleavage using one guide needs to be further explored.

Higher temperature helps the melting of the DNA duplex, facilitating the targeted double-stranded DNA cleavage by TtAgo^2^. We then tested the effect of elevated temperature on plasmid cleavage. We found that CpAgo with paired gDNAs could function optimally at 37 °C and that increasing the temperature to 41 °C did not lead to higher efficiency. However, a slight elevation of cleavage efficiency was observed when single guide was used (Supplementary Fig. S10a).

Since pAgos have no helicase activity, cleavage of the double-stranded DNA mediated by CpAgo and IbAgo is likely dependent on local sequence context. We generated pUC19-HGC plasmid by cloning a GC-rich sequence (100 bp, 55% GC content, Table S2) into pUC19 vector. Target plasmids pUC19-HAT and pUC19-HGC are identical except for the sequences flanking the gDNA-targeting site (AT-rich or GC-rich, Supplementary Fig. S10b). Using CpAgo and the paired gDNAs f1 and r1, the cleavage of pUC19-HAT is much more efficient than pUC19-HGC (Supplementary Fig. S10c), highlighting the importance of GC content of sequences flanking the gDNA-targeting sequence. As the negative supercoiling facilitates the unwinding of the DNA duplex^12^, we asked whether CpAgo and IbAgo could cleave linearized plasmid. In contrast to supercoiled plasmid, CpAgo and paired gDNAs (f1 and r1) could not cleave linearized fragment efficiently (Supplementary Fig. S11), demonstrating that appropriate topology of double-stranded DNA molecule is essential for efficient cleavage by CpAgo, likely via its effect on DNA duplex unwinding.

In conclusion, we identified two novel pAgo proteins that cleave single- and double-stranded DNA molecules in a DNA guide-dependent, sequence-specific manner at 37 °C. While we are preparing our manuscript, two similar studies were deposited on bio-archive^13,14^, describing a pAgo from *Clostridium butyricum* (CbAgo) having similar activities as CpAgo and IbAgo. Interestingly, along with CbAgo, the CpAgo and IbAgo described here are phylogenetically closely related, belonging to the same genus *Clostridium* (Fig. 1a). These findings raise exciting possibility of developing novel tools for precisely editing DNA sequences.

## Supplementary information

Supplementary information

Supplementary Table S2

## Supplementary information

**Supplementary Information** accompanies the paper at (10.1038/s41421-019-0105-y).
