# Supplementary material for: Argonaute proteins from human gastrointestinal bacteria catalyze DNA-guided cleavage of single- and double-stranded DNA at 37 °C
Source: Cell Discov. 2019 Jul 30;5:38. doi: 10.1038/s41421-019-0105-y (PMC6796838; doi:10.1038/s41421-019-0105-y)
Supplement: Supplementary file 1 — Supplementary information [file 41421_2019_105_MOESM1_ESM.pdf]

SUPPLEMENTARY INFORMATION

Table S1. Candidate pAgo genes

Fig. S1. CpAgo and IbAgo contain the catalytic DEDD tetrad.

Fig. S2. Sanger sequencing analysis of 100 nt ssDNA cleavage products using either 5’-P or 5’-OH gDNA.

Fig. S3. Cleavage activity of CpAgo and IbAgo on ssRNA substrate.

Fig. S4. Buffer optimization for ssDNA cleavage of CpAgo and IbAgo.

Fig. S5. Electrophoretic mobility shift assay (EMSA) showed the binding capacity between CpAgo or IbAgo with either 5’-P or 5’-OH gDNA.

Fig. S6. Permutations of the first nucleotide on the 5′ end of the guide are tolerated by CpAgo and IbAgo targeting ssDNA substrate.

Fig. S7. Target ssDNA cleavage activity of CpAgo and IbAgo loaded with mismatched gDNA.

Fig. S8. Effect of Mn^{2+} concentration on pUC19-HAT cleavage of IbAgo and CpAgo.

Fig. S9. Sanger sequencing analysis of pUC19-HAT cleavage products by CpAgo using one gDNA (f1).

Fig. S10. CpAgo functions optimally targeting sites flanked by high AT context at 37˚C.

Fig. S11. IbAgo and CpAgo could not cleave linearized plasmids at 37˚C.

Material and Methods

SUPPLEMENTARY INFORMATION, Table S1. Candidate pAgo genes

Organism	GeneID
Bacteroides sp.	BSHG_2835
Bacteroides sp.	HMPREF0106_01766
Bacteroides ovatus	CUY_4608
Bacteroides eggerthii	BACEGG_02178
Bacteroides fluxus	HMPREF9446_03003
Bacteroides cellulosilyticus	HMPREF1062_00677
Bacteroides fragilis	HMPREF1079_01015
Bacteroides fragilis	HMPREF1080_01078
Bacteroides ovatus	HMPREF1069_00739
Bacteroides fragilis	HMPREF1203_01196
Parabacteroides merdae	PARMER_00714
Parabacteroides goldsteinii	HMPREF1076_00757
Clostridium bolteae	HMPREF1095_05475
Clostridium bolteae	HMPREF1096_05797
Clostridium butyricum	WP_045143632
Clostridium perfringens	HMPREF9476_00452
Acinetobacter sp.	HMPREF0014_00504
Intestinibacter bartlettii	CLOBAR_02645
Alistipes putredinis	ALIPUT_02617

a

	D	E	D	X
RsAgo	VVGM**G**LAEL	ECEY**E**GYSD	VFHA**H**RPLK	IFYS**E**RIAE
eAgo	FLGA**D**VTHP	QHRQ**E**IIQD	IFYR**D**GVSE	AYYA**H**LVAF
PfAgo	IIGI**D**VAPM	EQRG**E**SVDM	LLLR**D**GRIT	VHYA**H**KFAN
MpAgo	YIGI**D**LSHD	LELN**E**KMNL	FILR**D**GRFI	LHIA**N**KVAL
MjAgo	IMGL**D**TGLG	GAPG**E**RLHL	LFLR**D**GFIQ	IHYA**D**KFVK
TtAgo	AVGF**D**AGGR	AQAG**E**RIPQ	LLLR**D**GRVP	LHLA**D**RLVK
NgAgo	FIGI**D**VSRS	PQLG**E**KLQS	VIHR**D**GFMN	TAYA**D**QAST
CpAgo	FVGL**D**VGTR	EQNG**E**KINT	VIHR**D**GFSR	TGYA**D**KICK
IbAgo	YIGL**D**VCRE	-QSG**E**KIQI	VFHR**D**GINR	TYYA**D**LSSI
CpAgo-CM	FVGL**D**VGTR	EQNG**E**KINT	VIHR**A**GFSR	TGYA**D**KICK
IbAgo-CM	YIGL**D**VCRE	-QSG**E**KIQI	VFHR**A**GINR	TYYA**D**LSSI

b

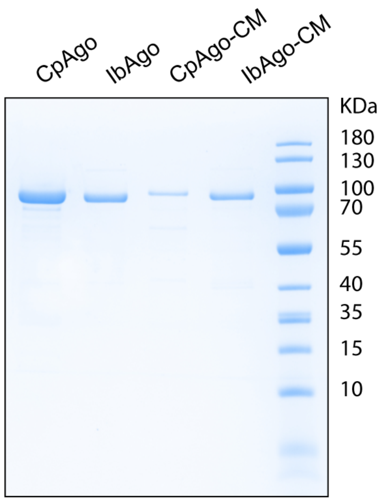

Supplementary information, Fig. S1. CpAgo and IbAgo contain the catalytic DEDD tetrad. a Multiple sequence alignment of the partial PIWI domain from CpAgo, IbAgo and their catalytic mutant form (CpAgo-CM and IbAgo-CM) with other characterized pAgo proteins. **b** SDS-PAGE analysis of CpAgo, IbAgo and their catalytic mutant form purified by Ni-NTA-affinity chromatography.

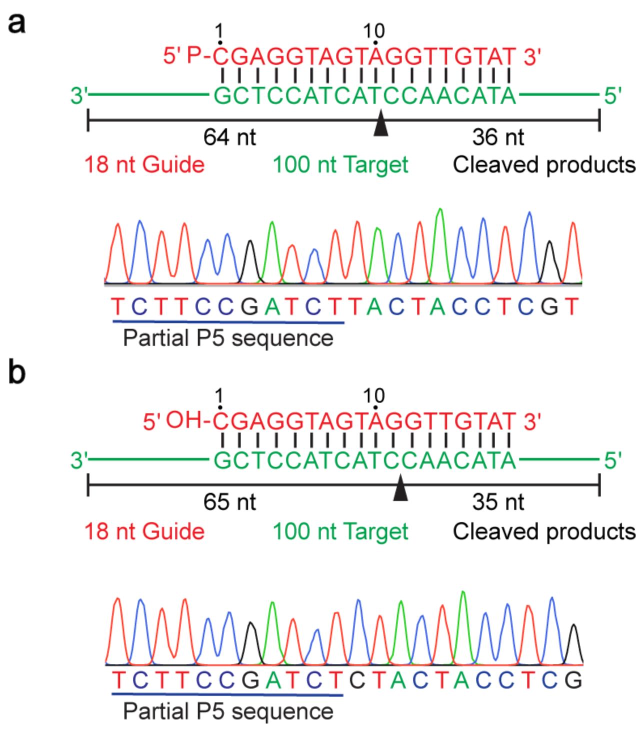

Supplementary information, Fig. S2. Sanger sequencing analysis of 100 nt ssDNA cleavage products using either 5’-P (a) or 5’-OH (b) gDNA. The black triangle indicates the cleavage sites, black lines indicate the corresponding cleavage fragments. The sequence directly downstream of the partial P5 sequence indicates the position of ssDNA cleavage site.

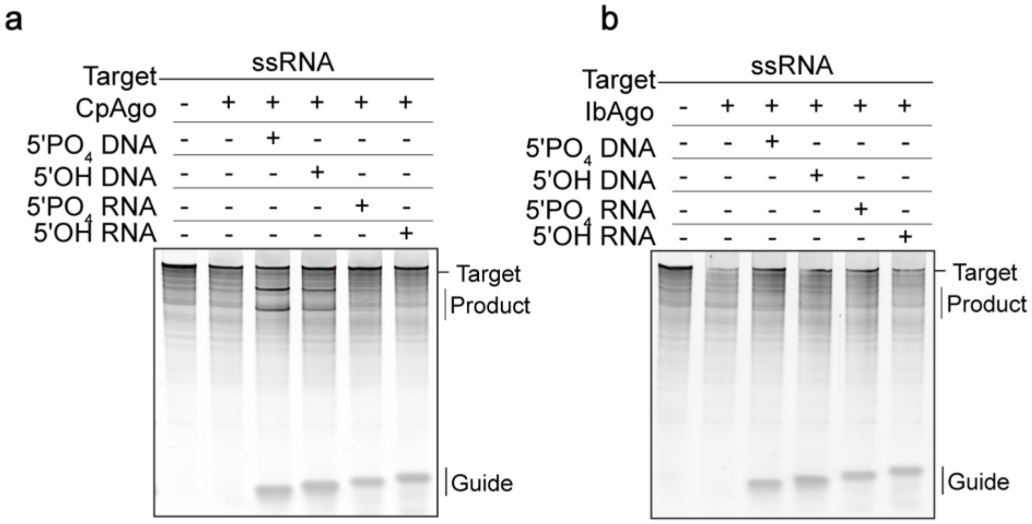

Supplementary information, Fig. S3. Cleavage activity of CpAgo and IbAgo on ssRNA substrate. CpAgo (**a**) and IbAgo (**b**) were pre-incubated with either 5'-P or 5'-OH DNA or RNA guides, followed by the ssRNA addition for 1 h. The cleaved products were analyzed by denaturing 15% PAGE.

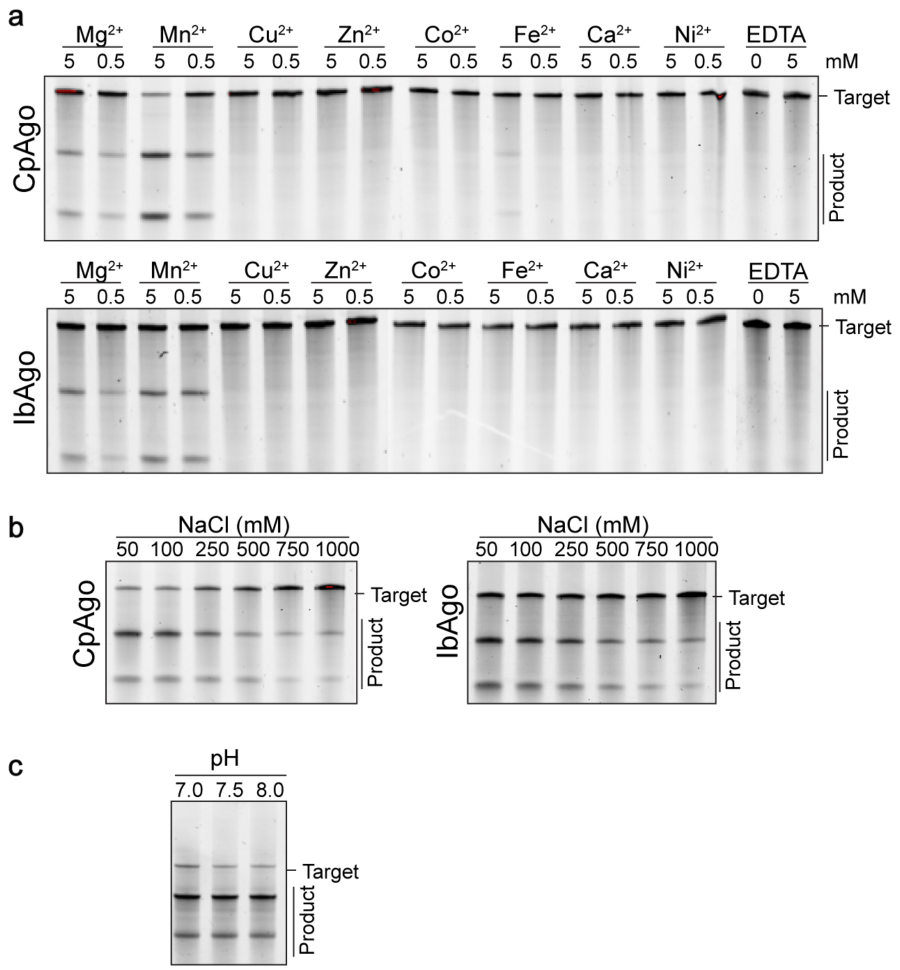

Supplementary information, Fig. S4. Buffer optimization for ssDNA cleavage of CpAgo and IbAgo. Effects of different metal ions (**a**), NaCl concentration (**b**), and pH values (**c**) on ssDNA cleavage efficiency of CpAgo and IbAgo.

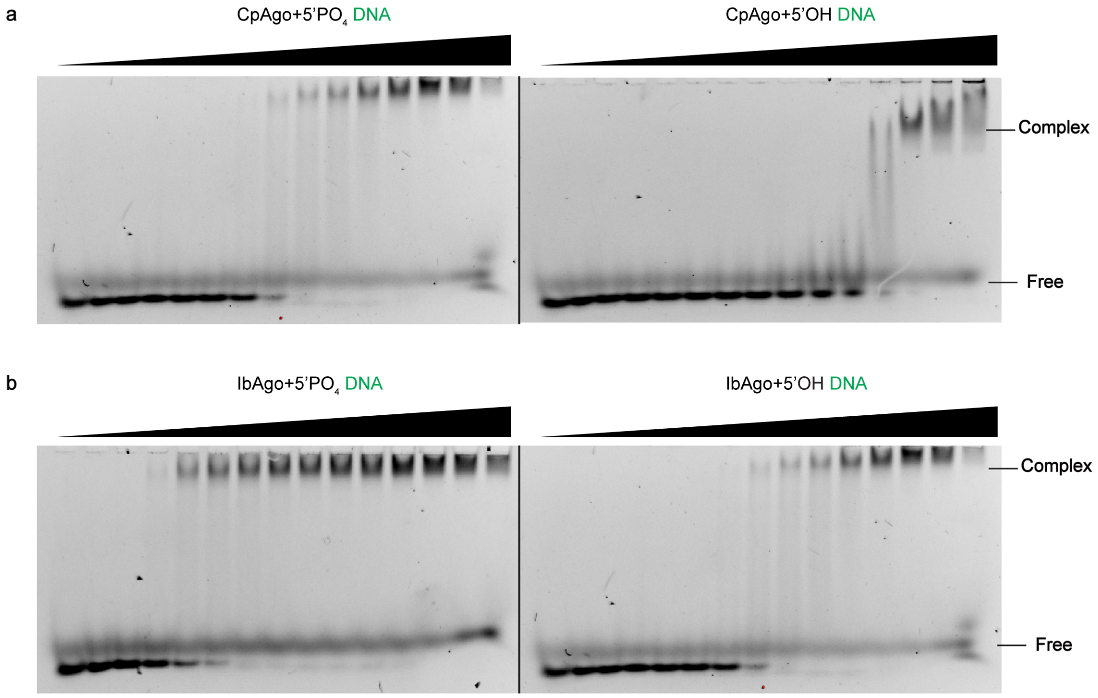

Supplementary information, Fig. S5. Electrophoretic mobility shift assay (EMSA) showed the binding capacity between CpAgo (a) or IbAgo (b) with either 5'-P or 5'-OH gDNA. Various concentrations (0, 10, 20, 40, 80, 160, 320, 640, 1280, 2560, 5120, 10240, 20480, 40960 and 81920 nM) of CpAgo or IbAgo were incubated with the same concentrations of 5'-P or 5'-OH gDNA labeled by 6-carboxyfluorescein (6-FAM) at 3' end, followed by a 6% native acrylamide gels analysis.

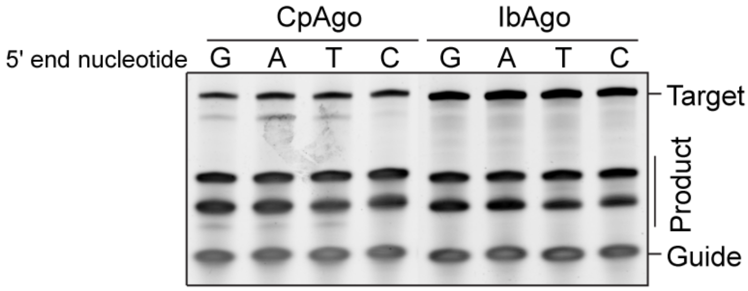

Supplementary information, Fig. S6. Permutations of the first nucleotide on the 5’ end of the guide are tolerated by CpAgo and IbAgo targeting ssDNA substrate.

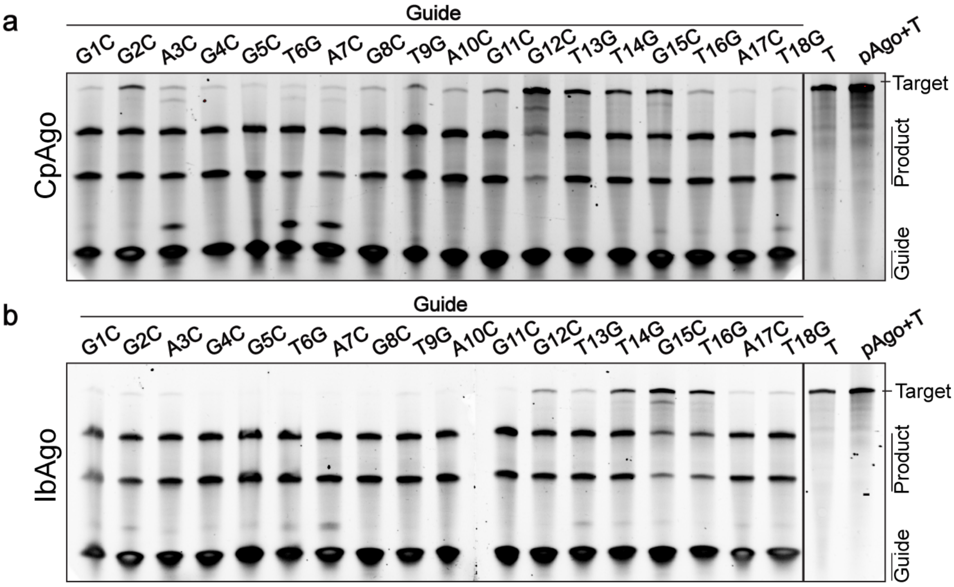

Supplementary information, Fig. S7. Target ssDNA cleavage activity of CpAgo (a) and IbAgo (b) loaded with mismatched gDNA.

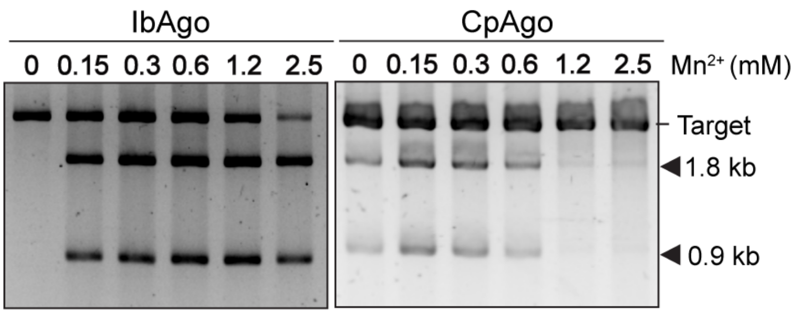

Supplementary information, Fig. S8. Effect of Mn^{2+} concentration on pUC19-HAT cleavage of IbAgo and CpAgo. IbAgo or CpAgo were pre-assembled with 5'-P gDNA pair (f1 & r1) and then performed plasmids cleavage (pUC19-HAT) for 2 h at the indicated $MnCl_2$ concentrations, followed by ScaI digestion for another 2 h and 2% agarose gel electrophoresis.

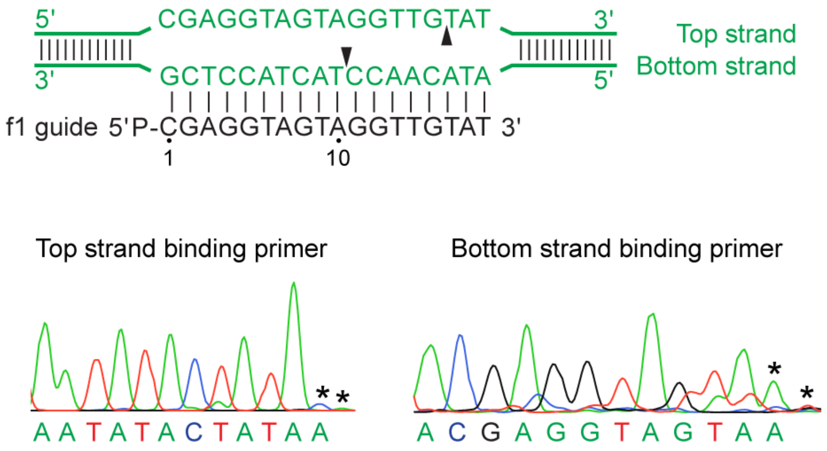

Supplementary information, Fig. S9. Sanger sequencing analysis of pUC19-HAT cleavage products by CpAgo using one gDNA (f1). The position of cleavage site is indicated by the termination of primer extension in the sequencing reaction. Sequencing artifacts are shown with an asterisk above the corresponding peaks.

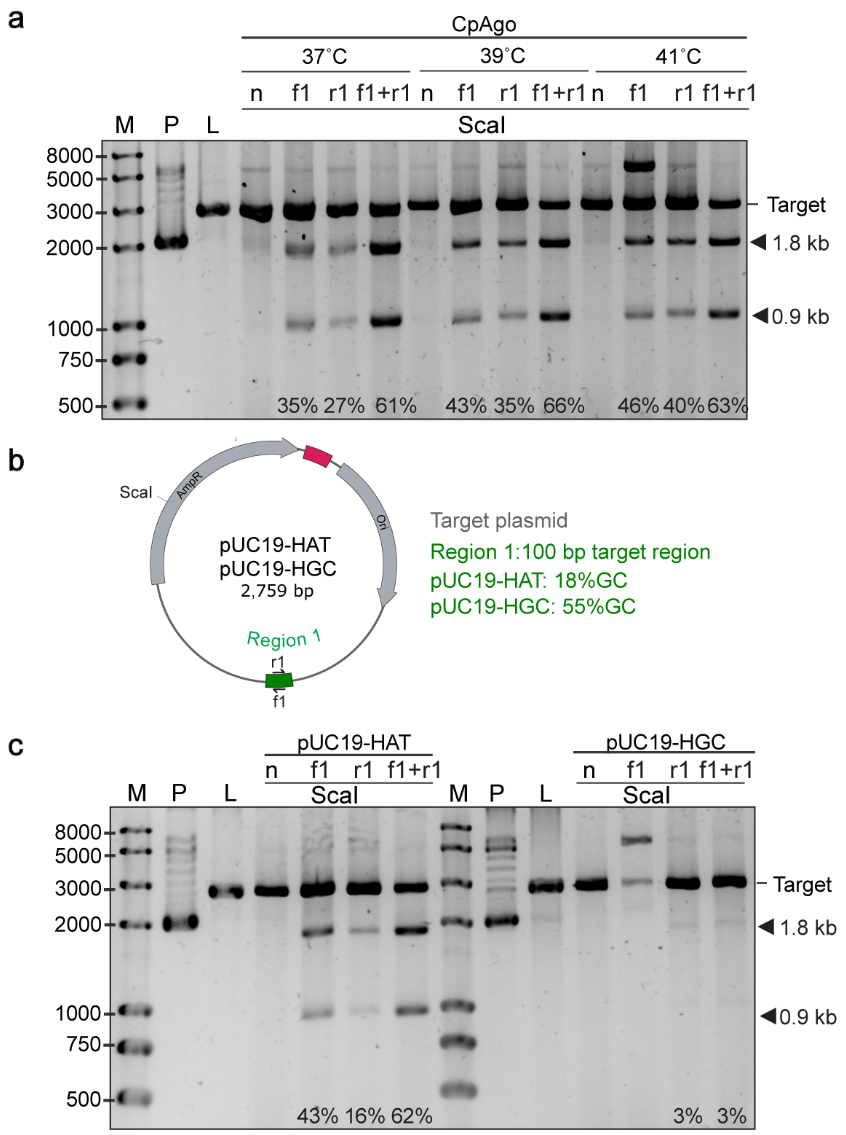

Supplementary information, Fig. S10. CpAgo functions optimally targeting sites flanked by high AT context at 37°C. a Effect of temperature on plasmids cleavage by CpAgo. CpAgo was pre-assembled with 5'-P gDNA and then performed plasmids cleavage (pUC19-HAT) for 2 h at the indicated temperatures. **b** Plasmids pUC19-HAT and pUC19-HGC contain a 100 bp target region with a GC content of 18% or 55%, respectively, as indicated in green. **c** CpAgo performs less efficiently when the target sites are flanked by high GC context (55% GC content). CpAgo was pre-assembled with indicated 5'-P gDNA before substrate pUC19-HAT or pUC19-HGC addition. The cleaved products were recovered by ethanol precipitation method, followed by ScaI digestion for 2 h and analyzed by 2% agarose gel electrophoresis.

The percent cleavage is shown below each lane. M: DNA ladder (Trans2K Plus II, Cambridge Reagents Limited). P: supercoiled plasmids. L: linearized plasmid. n: forward and reverse guides without sequence complementarity with target sequence. f1: forward guide. r1: reverse guide. f1+r1: forward and reverse guide.

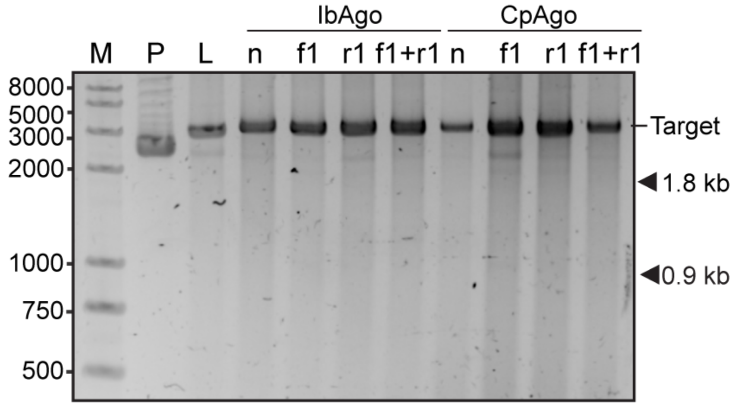

Supplementary information, Fig. S11. IbAgo and CpAgo could not cleave linearized plasmids at 37˚C. pUC19-HAT was first linearized using ScaI, and then incubated with IbAgo and CpAgo pre-assembled with 5'-P gDNA for 2 h. M: DNA ladder (Trans2K Plus II, Cambridge Reagents Limited). P: supercoiled plasmids. L: ScaI-digested plasmid. n: guides pair without sequence complementarity with target sequence. f1: forward guide. r1: reverse guide. f1+r1: forward and reverse guide.

SUPPLEMENTARY INFORMATION, Materials and Methods

Sequence alignment

For the active site residue sequence alignment, sequences from *Homo sapiens* Ago 2 (eAgo, NP_036286), *Thermus thermophilus* Ago (TtAgo, WP_011174533), *Rhodobacter sphaeroides* Ago (RsAgo,WP_044249552), *Marinitoga piezophila* Ago (MpAgo, WP_014295921), *Pyrococcus furiosus* (PfAgo, WP_011011654.1), *Methanocaldococcus jannaschii* (MjAgo, WP_010870838.1), *Natronobacterium gregoryi* (NgAgo, AFZ73749.1) were aligned with the ClustalW2. Only residues near the DEDX catalit tetrad were displayed for clarity.

Protein expression and purification

The CpAgo (WP_080564575.1; *Clostridium perfringens* stain WAL-14572) and IbAgo gene (WP_007287731.1, Intestinibacter bartlettii strain DSM 16795) were codon optimized for expression in *E. coli* and inserted into a pET28a expression vectors in frame with the N-terminal His tag. Catalytically mutants were introduced by site-directed mutagenesis using Q5 Site-Directed Mutagenesis Kit (NEB) and verified by DNA sequencing.

CpAgo, IbAgo and their catalytically mutant proteins were expressed in *E coli* strain Rossetta (DE3). For protein expression, cells were grown overnight at 37℃ in LB medium containing 20 ug/mL kanamycin. Then cells were transferred into fresh LB (1:100 inoculation) until an OD_{600} of 0.8 was reached. Then cultures were cooled down to 16ºC supplemented with 0.1 mM IPTG (isopropyl β-D-1-thiogalactopyranoside) at 16ºC while shaking for 18 h. The cells were collected by centrifugation and lysed by sonication (200W, 3s on/3s off on ice for 10 min) in Ni-NTA chromatography buffer A (50 mM Tris-HCl pH 7.4, 500 mM NaCl, 20 mM imidazole). Cell pellet obtained by centrifugation at 15,000 g at 4ºC for 30 min and the supernatant was loaded onto HisTrap HP colume (GE Healthcare). The column was washed with buffer A with 5 column volumes. Then the proteins were eluted with buffer A containing 300 mM imidazole, followed by dialysis overnight (~ 12–16 h) against 2 L of buffer (50 mM Tris-HCl, pH7.4, 500 mM NaCl) in the presence of thrombin protease (20402ES03, Yeasen) to cleave the His tag. Next the un-tagged

protein was applied to a second Ni-NTA affinity step. The flow-through was concentrated by ultrafiltration using Amicon 10K filter unit (Millipore) to a final concentration of 260 μM (CpAgo) or 100 μM (IbAgo). The purity of the protein was evaluated using SDS-PAGE (polyacrylamide gel electrophoresis) and visualized by Coomassie blue staining. Protein aliquots were flash frozen in liquid nitrogen and stored at -80°C.

Single stranded DNA (ssDNA) cleavage assay

Unless stated otherwise, ssDNA cleavage were performed at a molar ratio of 5:5:1 at 37°C. 3 μM CpAgo or IbAgo were premixed with 3 μM gDNA in reaction buffer containing 20 mM Tris-HCl pH7.4, 50 mM NaCl, 5 mM $MnCl_2$, 2 mM DTT for 30 min. Then 0.6 μM 100 nt target ssDNA were added and incubated for another 1 h. The reaction was terminated by adding an equal volume of Gel Loading Buffer II (95% Formamide, 18 mM EDTA, and 0.025% SDS, Xylene Cyanol, and Bromophenol Blue). The cleaved products were analyzed by 15% denaturing PAGE, followed by SYBR Gold staining and visualization with ChemiDoc MP imaging system (Biorad). For Fig. 1c, all reactions were incubated at indicated temperatures using a PCR thermocycler (Veriti 96 Well Thermal Cycler, ABI). For ssRNA targeting cleavage, the ssRNA substrate was transcribed in vitro using T7 polymerase (transcription template for ssRNA was listed in Supplementary Information, Table S2) and purified with MEGAclear columns (ThermoFisher Scientific) and eluted in RNase-free water.

Determination of ssDNA cleavage site

The 100 nt ssDNA substrate was cleaved as described above using either 5' P or 5'-OH gDNA with IbAgo. The cleaved fragments were recovered from PAGE gels through diffusion in TE buffer overnight at 37°C followed by isopropanol precipitation. Then the adapters were added to the ssDNA fragments using Accel-NGS 1S Plus DNA Library Kit (Swift Biosciences) under the manufacturer Instructions. As the kit would add a low complexity polynucleotide tail upstream of the P7 sequence with an average length of 8 bases to the 3' end of the fragment (the shorter fragment in this case), only the longer fragment downstream the P5 sequence at 5' end could indicate the cleavage site. Next, the product with adapters were cloned to the pEASY-Blunt Zero vector by TA cloning (Transgene) and 5 clones were sequenced for each cleaved product using either 5'P or 5'-OH gDNA.

Double stranded DNA cleavage assay

6 μM CbAgo was premixed with 12 μM of forward or reverse gDNA separately in reaction buffer containing 20 mM Tris-HCl, pH7.4, 50 mM NaCl, 0.15 mM $MnCl_2$, 2 mM DTT for 30 min at 37. Then the two separate reactions were mixed followed by addition of 100 ng target plasmids. The cleaved products were recovered by ethanol precipitation method and a subsequent digestion using either ScaⅠ-HF(NEB) or XbaⅠ (NEB) in Cutsmart buffer (NEB) for 2 h at 37ºC. The cleaved products were mixed with 6×DNA loading dye (MCLAB) followed by a 2% agarose gel electrophoresis and EB staining. IbAgo was treated the same as CpAgo except the reaction buffer containing 20 mM Tris-HCl, pH7.4, 50 mM NaCl, 2.5 mM $MnCl_2$ and 2 mM DTT.

EMSA assay (electrophoretic mobility shift assay)

10 nM 5'-P or 5'-OH guide containing a 3' FAM label were incubated with various concentrations of CpAgo or IbAgo in the reaction buffer (GS009, Beyotime) at 37℃ for 1h. Next, 10×loading dye was added to the reaction mixtures followed by a 6% native PAGE electrophoresis buffered with 0.5×TBE. Signal was collected and visualized using ChemiDoc MP imaging system.
